# The COVID-19 Pandemic Strain: Teleworking and Health Behavior Changes in the Portuguese Context

**DOI:** 10.3390/healthcare9091151

**Published:** 2021-09-03

**Authors:** Teresa Forte, Gonçalo Santinha, Sérgio A. Carvalho

**Affiliations:** 1 Department of Social, Political and Territorial Sciences, University of Aveiro, 3810-193 Aveiro, Portugal; teresaforte@ua.pt; 2 GOVCOPP, Department of Social, Political and Territorial, University of Aveiro, 3810-193 Aveiro, Portugal; 3 Hei-Lab: Digital Human-Environment Interaction Lab, School of Psychology and Life Sciences, Lusófona University, 1749-024 Lisbon, Portugal; 4 Centre for Research in Neuropsychology and Cognitive and Behavioural Intervention (CINNEIC), University of Coimbra, 3000-115 Coimbra, Portugal; sergio.andcarvalho@gmail.com

**Keywords:** COVID-19, coronavirus, SARS-Cov-2, teleworking, physical exercise, health policies

## Abstract

The COVID-19 pandemic has forced a societal essay, based on thorough measures of individual and communitarian protection, ranging from compulsory social distancing to quarantine. Following WHO recommendations, more or less strict policies were adopted by governments worldwide in order to mitigate public health risks. In Portugal, the first state of emergency was declared on 18 March 2020 and renewed until 2 May 2020. During this time, most citizens stayed in quarantine with practical implications regarding their work and daily activities. This exploratory study, conducted within the pandemic crisis context in Portugal, intends to grasp specificities of the adaptation to the lock down and social isolation/distancing measures, concerning, specifically, teleworking conditions and physical activity practice. Data was collected from March to May 2020 through an online survey from 1148 participants of different age groups and literacy. Considering that COVID-19 features a mutual feedback loop of disease and social dynamics—governmental measures, civic adjustments, and individual coping—to know more about what was featured, the first wave may provide some cues to ensure a more efficient co-operation among social actors and, ultimately, tailor better public policies towards teleworking, online distance learning, and the promotion of healthy behaviours.

## 1. Introduction

The implementation of measures to contain and delay the spike in infection of COVID-19 has resulted in major changes in both work and social lives, allowing us to test, in a natural setting experiment, different societal iterations.

**Figure 1:**
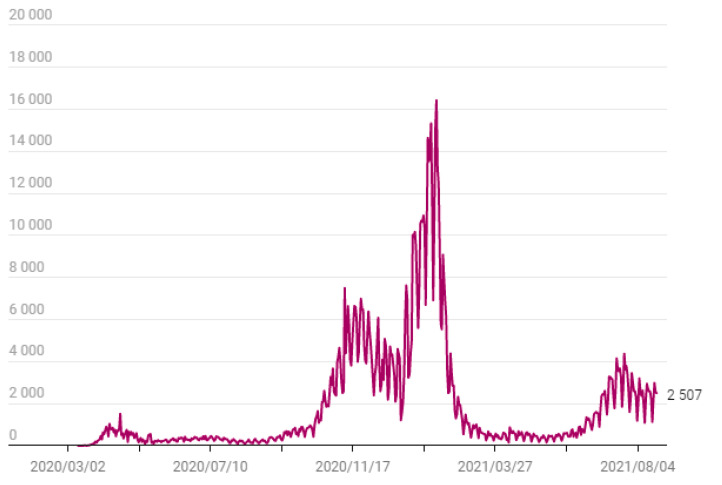
Evolution of new cases per day in Portugal. Source: https://expresso.pt/coronavirus/20-0-2021-Covid-19, accessed on 16 May 2020.

Although symptoms of COVID-19 are similar to those of other strains of coronaviruses (e.g., fever, dry cough, fatigue) asymptomatic individuals are able to spread the virus [1].The global outbreak of COVID-19 has thus prompted most countries to implement an array of measures to contain or delay the spread of the virus, from self-isolation or quarantine to public health guidance (e.g., hand washing, respiratory etiquette, social distancing) [2]. Although most countries have advised their citizens who display symptoms to self-isolate for 7–14 days, and practice social distancing to those without symptoms, the implementation of overall top-down governmental measures have differed according to each country (e.g., [3,4]). In Portugal, the first two confirmed cases of COVID-19 were reported on 2 March 2020 [5]. On the 18 March the President declared the state of emergency (President Decree no. 14-A/2020), which was consecutively renewed until 30 April with a positive impact in the number of new cases per day in this first phase, as evidenced in Figure 1.

During this first period, several measures were taken which impacted work, economic, and social spheres (see [6] for an in-depth description). As the expected number of cases of an infectious disease such as COVID-19 is directly generated from contact with an infected person, social distancing is usually used as a measure to curb the spread of the disease [7]. Accordingly, one of the most poignant ideas, with unprecedented worldwide application, was the sudden adaptation and shift of the workforce into a telework format.

Epidemic models seem to project that telework is indeed critical in buffering the overall burden of COVID-19 on the population [8], and companies expect it to be an essential component in the efforts to mitigate the pandemic [9]. With the implementation of telework, allied to the quarantine measures, expectedly comes an increase in sedentary life, as well as its widely acknowledged negative health consequences [10,11].

In this article, we argue that the understanding of the ways in which teleworking will unfold and be adopted at a macro-scale level may benefit from knowledge on how individuals have adapted to it during current pandemic times with no preparation or prior training. This study, of an exploratory and descriptive nature, is thus guided by the following research question: “What featured the adaptation to tele-working and social isolation in the first lockdown?” More specifically, it sought to characterize some physical, practical, and emotional adjustments related to this shift and co-presence between home and work during the first lockdown within the Portuguese context. It also intends to explore the changes in health-related behaviours, particularly the practice of physical exercise as a way to mitigate pandemic effects.

## 2. Teleworking Background

### 2.1. Pros and Cons of Teleworking

Telework, also called telecommuting and remote working, was first outlined by [12] as an original response to urban sprawl, traffic, and scarcity of non-renewable resources. The idea gained particular momentum in the midst of a crisis of a different nature, the OPEC oil embargo and the subsequent energy scarcity and cost inflation, which added to the increasing concern over gridlocks in major urban centres. Work settings reorganization was thus seen as a measure that would promote environment sustainability in the long run. Over the years, the appeal concerning the environmental benefits of teleworking remained, namely reducing transport-related environmental pollution, congested cities, and fostering rural development, given that people could work for city-based companies [13].

In the 1990s and 2000s, much due to technological breakthroughs that were quickly widespread, it gauged a lot of attention as a new flexible form of work organization [14] that would become a major feature of working life in western society. What is more, the claim expanded from societal and environmental benefits to how individuals and organizations could gain from this new setting [15].

Despite all, this more flexible form failed to launch at a global scale, much due to managerial and executive resistance [16]. At this level, albeit recognizing the advantages of flexible work, occupational and industry constrains, such as the fear of cultural change, inequitable outcomes and a flunk in workers’ motivation kept the inertia. Accordingly, no substantial research further explored the topic up until recently within the context of pandemic compulsory measures. As a case in point, a brief search on 30 June 2021 by ‘telework*’ on SCimago Journal Rank (SCOPUS), the most commonly used scholar citation database in social sciences, shows a scarce interest until the 90s, a steady focus of 30 and 40 publications per year in this decade, a slight increase in the 2000s to 50 and 70, and a rise to 200 articles per year in 2020 and 2021.

Currently, teleworking is defined as the provision of service done at a distance, using online and telecommunication technologies [17], hence allowing workers to fulfil their roles and functions while keeping the connection with the employer [18]. The locale where the worker develops his activities and the use of information and communication technologies (ICT) are thus two nuclear elements of teleworking.

The economic pressure, competition, and unforeseeable changes in society and job market have challenged the traditional conception of employment, forcing organizations to adopt, up to a certain extent, flexible work practices [19]. Made possible by the advances in technology within the last decade, these practices, varying in magnitude and extent of application, are overall seen as offering a competitive edge to companies (thus being a common practice in many high-tech businesses and start-ups particularly concerned in attracting and retaining talent invested in innovation). Besides the contribution to environment-friendly and healthier cities, several advantages have been reported associated to teleworking, at an organizational and individual level [20].

An evident benefit is to reduce the costs and time spent in commuting while sparing workers from the inherent stress and tiredness with negative consequences for their physical and mental health [21]. These perks, with a clear positive impact on the daily routine, may contribute to a higher satisfaction and dedication towards work which are well known predictors of a higher productivity [18]. On the other hand, they reduce the demands of the dialectic public/private and work/family roles [22], such as schedule flexibility, reduced costs of overheads, and increase in productivity (e.g., [21,23]). It is also argued that teleworkers tend to enjoy of more free time for leisure and can easily reconcile work with family demands, being more available or, at least, more flexible to take care and give attention to children or elder family members. In addition, the employer can also be more competitive and reduce some costs, as in electricity and water, as well as those related to sick leaves and workers absenteeism.

Others have emphasized the considerable disadvantages of teleworking, such as social isolation [24], presenteeism (e.g., working longer hours, working when sick) [25], and blurred boundaries between work and home life [26], which can negatively affect psychological well-being [27] and overall family dynamics [28]. Reduced social interaction is the main problem given its potential in fostering sadness, solitude, and stress and subsequently reducing work motivation (especially for those who live alone). Mixing work and personal life can also cause entanglement and confusion [22], even more when there is an overlapping of physical spaces. In this regard, it can be substantially harder to deal with distractions at home than those that occur in a traditional workspace, thus requiring more self-discipline and time management skills to succeed in teleworking [29]. This is true in both ways, for those who get easily distracted or those who may find difficult to disconnect compromising their health and wellbeing [22].

On a different note, [18] refer that teleworking may also impair career progression because, when working remotely, workers are less on the radar for possible promotions. What is more, workers may feel less connected to the organization and miss the social contact and usual exchange with co-workers that can lead to fruitful collaborations [30]. In this regard, ref. [31] argues that personal interactions have a superior impact, particularly due to the enabled visual contact. Video calls and similar interactive devices fail to mimic this experience; hence, it is arguable that new technologies foster a particular type of distance among workers.

### 2.2. Teleworking during the Pandemic

The effective implementation of teleworking as a means to mitigate the seemingly unavoidable economic impact of the COVID-19 was especially relevant to countries such as Portugal, in which positive signs of economic growth were appearing prior to the pandemic outbreak. Through covering at least the functions compatible with working at a distance, the benefits are evident since it allows workers to keep their jobs and allows firms to continue developing their activity, reducing the economic burden [31].

However, this measure was implemented without specific regulations, only based on a general agreement on teleworking of 2002, drawn on a different stage of ICT development and EU-based policies and directives regulating work, in general, and assuming by default that the same provisions would apply. Among these, are: EU Directive 2003/88/CE, about working time schedules; EU Directive 89/391/CEE on work health and hygiene; EU directive 2019/1158 about dealing with professional and familiar life and; EU Directive 2019/1152 on transparent and predictable work conditions [32]. The highlight goes to general rights, such as the voluntary nature of the work; respect for privacy; data protection; health and safety measures.

Only in June 2020, an autonomous framework, aimed at informing a possible European-based directive on digitalization, was put forth covering four specific areas: digital competence and job security; connection and disconnection modalities; artificial intelligence and human control; respect for human dignity and vigilance. The emphasis on these areas provides cues about the main concerns of conducting work activities with such dependence on ICT. Furthermore, a few recommendations are drawn so as to protect the workers’ rights on these conditions, starting with being informed about all the matters regarding equipment, working hours (normal and extraordinary), responsibilities, and costs. Other important provisions regard the costs being completely covered by the employer; the extraordinary hours reimburse; the right to sick leaves and, very importantly, an efficient and fair measurement and monitoring of working hours so as to protect workers from the risk of presenteeism.

Besides not knowing the impacts and effects on a wide array of indicators in the long-run, either related to productivity and financial aspects, the individual and social coping to the hypothetical dissemination of teleworking is also uncertain.

The literature puts forth two coping strategies: “integration” and “segmentation”/“separation” [33]; both are based on how individuals redraw cultural boundaries around “work” and “home” when these overlap, as occurs in a teleworking format. These coping strategies, although generalist, provide a conceptual lens to the practicalities of accommodating the co-presence of these two settings with the ethical and values with which they are imbued [34].

In this regard, a separatist approach features the co-presence of “work” and “home” by adhering to strict temporal regimes as expressed in fixed office hours and closed-door spaces. Thus, symbolically as well as practically, “work” and “home” are kept apart. An integrative approach, on the other hand, tends to be more flexible and is likely to follow a more laissez-faire temporal regime, integrating domestic, personal activities (as physical exercise) and professional activities in common spaces [35].

Underpinning the coping strategies lies a fundamental element regarding the gendered division of household and childcare responsibilities [36]. Domestic inequalities are still a reality, particularly in countries with lower levels of gender equality and female empowerment [37,38] and, during the pandemic, they appear to have increased, especially amongst people with children [39]. More specifically, mothers reported a decrease in working hours and an increase in domestic and house care activities, as well as supervising children’s homework and didactic activities [39,40], with a negative impact on their wellbeing [36]. This is in line with the gendered expectations that remained the same and, despite the expansion of women’s roles in the last decade working outside the home, they are still expected to perform most of the domestic and care work [41].

What is more, gendered roles are prescriptive and proscriptive of attitudes and behaviour, and both have been evidenced, especially in the beginning of the pandemic, with women reporting more psychological distress and anxiety, and men reporting strength, more calm, and determination [42].

This forced experience on teleworking is perceived as an opportunity to catalyse “a wider adoption of teleworking practices also after the crisis” [42]. According to the European Foundation for the Improvement of Living and Working Conditions [43], more than three quarters of EU workers prefer to work from home, at least occasionally, even without COVID restrictions. Specifically, most EU workers indicate that they had a positive experience of teleworking and, albeit not exclusively, the most favoured option is to combine teleworking and on-site work.

However, the overlapping of leisure and working time, domestic and labour routines, as well as the ICT intensive use are known to impact health and wellbeing. The negative effects are mostly psychological pressure, stress, vision problems, anxiety, headaches, fatigue, sleep disorders, and skeletal muscle functions [43].

In order to counteract the physical and mental health impact of telework, and to promote overall healthy behaviours during the pandemic, public health communication should not only focus on messaging information strictly regarding COVID-19 infection and its mitigation (e.g., prevalence, progression, death rate, mitigation measures) but also health promoting behaviours related to the management of in-door time and physical exercise. Indeed, some have advised for the maintenance of physical exercise during lockdown (e.g., [44]), and it has been argued to help reducing the negative health consequences of COVID-19 quarantine [45].

### 2.3. Occupational Health in Telework: The Importance of Physical Activity

According to the World Health Organisation [46], a healthy workforce is crucial for social and economic development. The WHO’s report on occupational health states that there is a continuous two-way interaction between individuals and the physical and psychological working environment, as the latter may affect, positively or negatively, the worker’s health, and productivity is, in turn, disturbed by the person’s well-being. In view of this, in order to ensure occupational health in telework in the context of COVID-19, it is important to underline the health risks and benefits associated with the sudden and large-scale shift to telework, as well as the specific conditions that lead to better psychological and work outcomes [47].

Within the Portuguese context, a qualitative shift occurred in Health promotion initiatives, as evidenced in the official communication issued by The National Program for Physical Activity of the General Health Department (2020a) [42]. Aimed at counteracting the demanding restrictions, both resulting from spending more time at home collapsing routines and spaces as from being limited to enjoy public spaces, health authorities have been forceful in ensuing specific recommendations adapted to the circumstances. Very directive suggestions included: avoiding to seat or lie down for more than 30 min; reduce the time spent using technological devices; walk inside the house and conduct other physical activities; ‘invest in activities of cognitive stimulation (reading, puzzles); stretch and meditate as well as play with children [48,49].

This is backed up by WHO, suggesting 30 min of intense or moderated physical activity ([49]), particularly regarding older citizens [50,51], given their higher vulnerability to health problems and COVID-19. In this regard, aerobic home exercise has been advised, due to its fairly low complexity, low risk of injury, and high popularity [52].

Also, physical activity seems to be negatively correlated to cardiovascular disease and diabetes (e.g., [53]), which is especially noteworthy in the context of COVID-19, given that these constitute risk factors associated to respective severity and mortality (e.g., [53]). Additionally, exercise has been reported to positively impact anti-inflammatory response and reduce immunologic abnormality [54,55].

It is self-evident that physical activity has been impacted by the global efforts to mitigate the progression of COVID-19 infections [56]. In this social distancing phase, the type of physical activity should prioritize interiors or secure empty public spaces. Additionally, ref. [45] puts forth that people should practice physical exercise five to seven times a week, depending on the training intensity and modality (for example, if is resistance training it should be done two to three times a week, according to [57].

However, several obstacles may hinder the engagement of at-home physical exercise, namely the unavailability of training materials and equipment for moderate to intensive physical activity (particularly from those with a lower socio-economic level with less margin to acquire them), as well as difficulties in controlling training variables, such as adequacy of training exercises.

Notwithstanding the obstacles, one may argue that the disruption of normal life and routines, allied to the sudden official Public Health communication issued by governments and reinforced by all media, led to a salience of physical activity in peoples’ minds. Even though physical activity promotion and healthier lives are two common claims in western societies, the pandemic added a tone of threat and urgency to it, either as a way to reinforce the overall physical health or to mitigate the psychological impact of the quarantine measures.

In this regard, more fine-tuned research is needed to conclude the impact of the perception of public health messaging on the population´s adherence to governmental guidelines, including the appeal to physical exercise, as people tend to comply with governmental suggestions/orientations even when distrusting the government. This is particularly true in a time where information is not exclusively delivered directly by the institutions but rather mediated by both traditional and social media [58] with potential impact not only on compliance but also on mental health (e.g., [59,60]). In the context of COVID-19, studies suggest that using deontological moral advice when communicating public health advice (e.g., eliciting a sense of civic duty, ethical self-care) contributes to the engagement of behaviours that are helpful for health and wellbeing [61].

The ingrained notion of how important physical exercise is to physical and mental health found a more fertile ground because of the lack of parallel distractions.

Digital landscapes (with emphasis of YouTube and social media) played a quintessential role in this dissemination, fuelling a wide variety of online training offers, thus, expanding the outreach of gymnasium, sport clubs, and personal trainers. Recorded and live sessions, mimicking physical training, push good practices and physical activity support further, often on a daily basis [62], with the common denominator of being mainly home-based.

One may further argue that physical activity also contributes to mitigate the presenteeism and cognitive overload of connection, known to underpin physical and emotional exhaustion. In this regard, it is another aspect to take into account when drafting guidelines at EU level.

Drawing on data collected during the first locked down, the present work contributes to unveil key elements that may be considered in communication and public policies regarding teleworking and physical activity tailored to reach different segmented groups of the populations.

## 3. Materials and Methods

### 3.1. Participants and Procedure

Data was collected from 14 March 2020 to 2 of May through an online survey in google forms which was shared via institutional and personal contacts. There were 1148 participants who replied, 69.9% women (*n* = 802) and 30.1% men (*n*= 346). The sample includes five different age groups: until 18 years old (*n* = 8; 0.7%); 18–24 years old (*n* = 277; 24.1%); 25–39 years old (*n* = 261; 22.7%); 40–59 years old (*n* = 466; 40.6%); above 60 years old (*n* = 136; 11.2%). A substantial percentage of our sample has high education studies: nearly half is graduated at BSc level (*n* = 563; 49%), 19.8% at Master level (*n* = 227), and 7.1% has a PhD (*n* = 81). 15.9% (*n* = 182) has finished middle school and 8.3% (*n* = 95) completed 11° grade. More than half of the participants (*n*= 722, 62.9%) has a full-time job (40 h or more per week); 18.8% (*n* = 216) are students; 8.1% (*n* = 93) are retired; 4.4% (*n* = 51) work part-time jobs (16 to 30 h a week) and 44 (3.8%) are unemployed. Nearly half of the participants (*n*= 541; 47.2%) are married or living with a companion; 42.7% are single; 8.7% (*n* = 100) are divorced, and 16 (1.4%) are widowed. More than half (*n* = 589; 51.3%) have children. Approximately 60% of the participants indicate that their youngest child is still under age.

### 3.2. Questionnaire

The questionnaire applied was made available online and included an informed consent describing the study, the aim and topics included and informing participants about the confidentiality of their answers. Only a positive reply would allow to proceed to other items related to topics out of the scope of the present article (factual knowledge, perceptions, attitudes, and behaviours towards the virus, its transmission, and consequences), socio-demographic information, and the following sections used in the present study (Supplementary Materials):

Emotions: 5-point Likert scale items related to the emotional response felt in the last week (calm, nervous, sad, relaxed, and preoccupied).

Teleworking and physical activity: 20 items related to teleworking (physical conditions, technological dimensions, and communication) and 17 items concerning online and physical activities.

## 4. Results

### 4.1. Adaptation to Teleworking

The professional activities of most of the participants, 81.1% (*n* = 828), are compatible with teleworking, which is exclusively conducted from home. Interestingly, 34.2% consider that their professional routine has not changed, suggesting that there were sufficient elements in this period to maintain a perception of constancy. This may result from the fact that professional activities, nowadays, rely much more on online communication and technological media than on physically grounded activities. Hence, even though the context of work differed, the work process itself, at large, did not suffer significant changes.

An aspect reported as being different was the time spent in work-related activities mediated by ICT. In this regard, 37.3% of the participants indicate spending more time online or using some ICT (e.g., computer; telephone); 26.4% indicate attending to more meetings and 34.6% to work for longer hours.

Concerning the financial practicalities of this shift, 79.4% of the participants did not receive any reimbursement for extra expenses and 74.5% were not payed for extra hours. What is more, at the time, 18.8% did not even know if they would be reimbursed.

The working hours and financial provisions appear to be at odds with the applicable European directives on this issue, regarding, in particular, the reimbursement for any extra costs related to teleworking and communications and the appropriate compensation for extraordinary working hours, particularly onerous for those participants who report working longer hours. Although these shortcomings may be understood in the light of the lack of national-based regulation on teleworking, they strengthen the need to reinforce public policy on this matter, at EU and national levels, as is currently ongoing based on the independent framework of digitalization rights (see SOC/660–EESC-2020-05278-00-00-AC-TRA (EN) 2/18).

As concerns, one of the key factors of teleworking—its physical space—among the surveyed, 72.2% (*n* = 594) were developing their activities in common and shared spaces, such as the living room (44.6%); the bedroom (19.6%) and the kitchen (4.5%). Only 27.8% had a specific room in the house dedicated solely to work without overlapping with other family dynamics, which is suggestive that the majority of our participants faced one of the most problematic issues in teleworking that is the physical blurred boundaries between work and home life [26]. This is even more impactful considering that 47.2% were in a relationship, 51.3% had children, of who 61% were under 18 and living at the house.

Perceived as one potential disadvantage of teleworking [22] the shortcomings of the co-presence between work and home were particularly noteworthy in the context of COVID-19, given that, due to large-scale schools closing, parents not only have to juggle work and family life, but also manage children’s home schooling.

Interestingly, in line with what was found in [36], the toll was felt heavier by the women. As shown in Table 1 below, when asked about the emotions felt in the past week, men clearly reported more positive emotions than women, including feelings of calm and relaxation, and, in contrast, women differed significantly from men in showing more negative emotions, including nervousness, sadness, and preoccupation. A one-way ANOVA (data not shown) shows that there are significant differences between the groups in all the emotions assessed.

This strengthens the findings of [41] where women reported higher psychological distress whereas men were apparently calmer and stronger. These results may be influenced by the expected gendered display of emotions but also due to extrinsic pressures, since, in general, women were overall more burdened with more domestic and house care activities, as well as supervising children homework and didactic activities ([39,40]), with an expected negative impact on their wellbeing [36].

The analysis of the emotional reactions during this period also showed that, comparing all ages, participants above 60 years old are those that, albeit at a higher risk of pandemic-related complications and more targeted by official communication, were feeling calmer (M = 3.54; DP = 1.06), more relaxed (M = 2.77; DP = 1.12), less preoccupied (M = 3.46; DP = 1.11) and less nervous (M = 2.40; DP = 1.12) than younger individuals. Sadness was the only emotion equally felt by all groups, appropriate to the loss and disruption felt at those times.

The overall concern about older individuals’ health vulnerabilities and risk of social isolation and higher emotional impact [48] is not corroborated in our sample, with younger individuals feeling more negative emotions during these times. This may be related to the work-related uncertain processes and outcomes of the pandemic impact. Interestingly, students are the ones reporting higher levels of sadness (M = 3.12; DP = 1.13) whereas workers (62.9% of our participants have a full-time job and 4.4% a part time) report more nervousness and preoccupation, particularly part-time workers, the most psychologically distressed segment. Negative emotions in workers may also be aggravated by the fact that 40% of the participants work more hours than before at their work places. This result, besides not abiding by general regulations, is at odds with the more optimist view of teleworking as allowing workers to enjoy more free time for leisure [21] and is, in turn, in line with the risk of presenteeism [25] and overall negative impacts for the psychological well-being [27].

**Figure 2:**
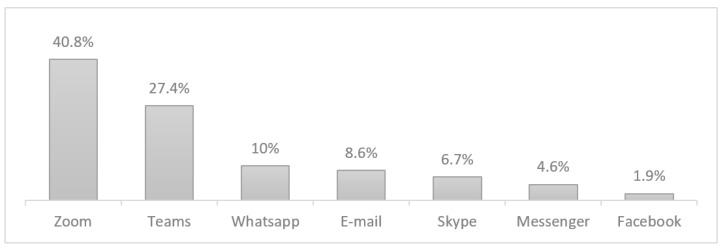
New ICT tools.

The work spillover during leisure hours is not, however, the only problematic issue. The non-verbal overload of digital interaction is known to not only fail at mimicking a healthier personal experience as to foster tiredness and irritability [31]. This is particularly evidenced in meeting platforms, such as Zoom or Microsoft Teams, in which increasing use is also corroborated in the present study. As shown in Figure 2 below, Zoom was the more frequent new ICT platform followed by Microsoft Teams. The remaining were already commonly used for communicating with teams and co-workers, especially e-mail (99.9% of the participants), followed by WhatsApp (56.4%) and Messenger (40.8%). Other studies have reported similar results, in which the use of and dependence upon social media platforms, such as Zoom, Microsoft Teams, and WhatsApp, to stay connected for work, education, and social purposes, have seen an exponential growth in users during that time (e.g., [63,64])

In this regard, 64.6% of our participants report not using ICT for leisure, suggesting their use as working or utilitarian tools. One of these utilitarian aims, besides work, is online shopping, with 45.8% of the participants reporting it as a common practice. For 23.1%, the frequency of on-line purchasing has increased during the pandemic that also brought a different choice of products (depicted in Table 2). Expectedly, considering the measures of social isolation and quarantine at place, there was a substantial increase in the acquisition of essential goods and foodstuffs. Gadgets and technology purchase also increased, probably due to the higher ICT use during these times for work and entertainment purposes. Interestingly, there was a fall in all of the other products, particularly clothes.

Among the 35.3% who actually use ICT for leisure, the interests and focuses are varied (see Table 3). Physical exercise classes and apps are the more frequent on-line based activities, and this interest and actual investment speaks favourably about the widespread dissemination of the importance of physical exercise. This in-home practice even surpassed the search for entertainment-based activities, as internet searches, movies and TV shows, and games.

### 4.2. Physical Activity

The interest in being physically active is not only evidenced by searching and purchasing related physical activity apps and classes online, but also by the fact that 70.1% of the participants were already active before the pandemics, 53.1% practicing a specific sport and 46.9% recreative and leisure physical activities.

Even though 54.1% report that the physical activity decreased with the pandemic, 27.7% were still practicing up to 3 times, 19.9% once a week, and 17.3% up to seven days a week, which is not so far from the optimal practice suggested in [45,57]. These regular habits are even more important considering that 54.2% of our participants work seated at the computer with the potential sedentarism and collateral psychological pressure, stress, vision problems, anxiety, headaches, fatigue, sleep disorders, and skeletal muscle functions [57]. Furthermore, there was a substantial decrease for younger participants (52%) and for participants above 60 years old (66%), which strengthens, even more, the governmental concerns in targeting this age in particular [50].

As expected, there was a shift in the place of physical practice and whereas 91.7% of these activities were practiced outside the house with the pandemics, only 20.2% of the participants were able to keep that routine. Moreover, 79.8% of the participants report to conduct their physical activities inside the house, suggesting an adherence to the message issued by governments and reinforced by all media concerning the practice of physical activity [56]; ICTs, in particular, digital landscapes such as YouTube, social media, and sites, appear to be of nuclear importance in the adoption of this practice mimicking a real life context of physical practice and connection [62] while 39.3% of the participants report following a regular routine nowadays.

Another evidence of the compliance of governmental indications is the difference between the role of group-based activities of physical exercise before (41.6%) and during the pandemic (3.9%). There was no change, however, in the percentage of participants exercising in the company of one more person. Despite the overall frequency decrease, one may argue that what changed for most of them was the adjustment to different routines since—up to a higher or lesser degree—they have started to practice inside the house and, more often, alone (73.1% of the participants in contrast with 33.8% prior to the pandemic).

The practice of physical exercise appears to be more frequent in participants with a master degree (81%) and a PhD (79%) and the least adopted by those with a compulsory education (55.8%). These results follow the widely acknowledged association of physical exercise with health behaviour and better health in general [65] being perceived by some authors as the single most important and constant influence in health preservation [66].

On one hand, it is argued that formal education fosters knowledge and values related with seeking and comprehending health-related information as well as acting upon it. By contrast, lower educated people are at higher risk of not engaging in the desirable levels of physical activity [67], which can also be linked to more material problems (such as housing general conditions and available space) or poor health experienced by older lower educated people.

Accordingly, public health communication should emphasize beneficial and low complexity exercises (as aerobic home) assessable to all segments of the population.

## 5. Conclusions

### 5.1. Implications

The COVID-19 pandemic has embodied a major challenge, not only for the health system, but also for services, firms, workers, and employers, due to the upswing suddenly experienced by remote working technologies. The spread of teleworking and the use of technological platforms, in this context, has been considered essential to keep social distancing in workplaces and between employees and users/clients. Given the speed of change in result of political measures, services, and companies had very little time to put together a work at distance plan. Even though the COVID-19 pandemic and its mitigation methods have noticed, these past months, a gradual decrease in a number of countries concerning social distance, the extensive use of teleworking is expected to continue. As recently stated by the European Parliament Committee on Employment and Social Affairs, “the extensive use of telework poses a number of challenges and requires a re-think of the way work is performed, coordinated, and regulated” ([68], p. 14), bearing in mind its positive and negative impacts. On this, several hazards to the health of teleworkers have been highlighted in literature (see inter alia [69]), namely physical (e.g., awkward postures, repetitive movements, and long periods of continuous work, increased rate of physical inactivity, and sedentarism) and psychosocial (e.g., sleeping disorders, work-related stress, and social isolation) ones.

If COVID-19 events have transformed the working conditions and modified the employer-worker-user/client relationships, making telework unlikely to return to pre-pandemic levels, it is essential that policymakers, services, and firms realize the challenges associated with this phenomenon, building knowledge to provide the basis for change, improvement, and, accordingly, promote generative learning from research. This study, conducted within the COVID-19 crisis context in Portugal, intended to grasp specificities of the adaptation to the lock down and social distancing measures, in what concerns specifically teleworking conditions and physical activity practice.

From this study, it is possible to derive some findings with potential implications for the immediate and post-pandemic settings. First, the workload and time spent in teleworking were higher than in the physical format, i.e., before the pandemic. Besides confirming the risk of presenteeism (foreseen as disadvantage of this format) it reinforces the need to draft clear and encompassing regulations and policies protecting the workers from this probable spill over.

Our results also unveiled a problem related to the workers’ personal sphere, that is, the lack of a specific space at home exclusively for work. The overlapping of spaces and blurred boundaries between work and home life is known to cause entanglement and confusion as well as be much more demanding in self-discipline and time management. Even though it is harder to tackle this issue from a public policy viewpoint, it may be mitigated at an organizational level: team-leaders and employees need to be briefed and prepared in the most co-constructive ways to conduct work in these different and heterogeneous conditions. Under a common teleworking policy, trainings, specific performance criteria and weekly check-ins to gauge their experience and address any concerns should be adopted. A people-oriented mind set would be beneficial, acknowledging and managing, as much as possible, the pressing anxiety and stress that may result from these conditions.

On the other hand, this research also suggests that women were subjected to more emotional stress and impact on psychological wellbeing. This requires a tailored approach to raise awareness about expected gendered biases while empowering women to assert and define a more balanced distribution. Given that this is a structural societal issue, it may be more effective if put forth and advocated by public or organizational policies.

The same concerns apply to the wide use of ICT, also corroborated here, known to induce a cognitive overload with a negative impact on wellbeing and physical health. Efforts in tailoring occupational health programs and training should be put in motion and enforced by public policies. This may also include the emphasis, already noticeable, of the perks and necessity of physical activity, no longer seen as a hobby but as a complementary part of a work routine. This study indicates that, despite the difficult conditions and adverse times, there was an effort to continue to practice physical activities (also evidenced in the search for related online classes and apps), which speaks favourably of the receptivity to Health communication and individual predispositions.

In addition, the lack of reimbursement for extra work time or equipment, at par with the workers’ unawareness of their rights and what they are entitled to, is indicative of the urgency in drafting regulations and legislations at the European and national level specifically covering telework. Considering the gradual shift towards flexible work practices, these regulations should be well-known by the workers.

### 5.2. Limitations of the Study and Future Research Lines

The present study has two main limitations to be taken into account and frame the results interpretation. The first concerns its exploratory and descriptive nature, reflected both in the questionnaire design and in the analyses conducted which targeted only a description of general conditions and particular behaviours and practices of the participants. The second regards the non-probabilistic sampling method through institutional and personal contacts, which resulted in an over-sampling of highly educated individuals. In this regard, the results must be considered in the light of this particular WEIRD sample and national context.

Notwithstanding, considering the increasing role teleworking is playing in society, this study highlights some patterns that may inform further research and policy design particularly in the analysed context, worth to emphasize that public policies and co-operation among social partners are crucial to ensure that new, efficient, and welfare-improving working methods emerging during the crisis are maintained and developed once physical distancing is over. To maximize productivity and welfare gains inherent in the use of more widespread telework, governments should promote investments in the physical and managerial capacity of firms and workers to telework and address potential concerns for the workers’ health, well-being, and longer-term innovation related, in particular, to the excessive downscaling of workspaces.

## Figures and Tables

**Table 1:** Means and Standard deviation of emotions by gender.

		N	M	SD
Calm	Male	346	3.82	1.01
	Female	802	3.25	1.04
Nervousness	Male	346	2.25	1.10
	Female	802	2.89	1.15
Sadness	Male	346	2.60	1.16
	Female	802	3.12	1.19
Relaxation	Male	346	3.06	1.08
	Female	802	2.53	1.04
Preocupation	Male	346	3.28	1.11
	Female	802	3.74	0.99

**Table 2:** Online purchases before and during the pandemic.

	Before	During Pandemic
Essential goods and foodstuffs	26.9%	46.8%
Clothes	46.5%	9.4%
Cosmetics	5.4%	4.7%
Books	15.8%	7.4%
Gadgets/Technology	5.4%	31.7%

**Table 3:** Categories of on-line activities for leisure.

	N	%
Physical exercise classes and apps	107	27.30%
Internet searches (sites, YouTube)	103	26.40%
Movies and tv shows (Netflix, HBO)	98	25.10%
Games	71	18.20%
Cultural activities (cinema, theater, concerts)	50	12.80%
Social Media	35	8.90%

## Data Availability

Not applicable.

## Supplementary Materials

The following are available online at https://www.mdpi.com/article/10.3390/healthcare9091151/s1, Questionnaire.
