# Supplementary material for: The COVID-19 Pandemic Strain: Teleworking and Health Behavior Changes in the Portuguese Context
Source: Healthcare (Basel). 2021 Sep 3;9(9):1151. doi: 10.3390/healthcare9091151 (PMC8468513; doi:10.3390/healthcare9091151)
Supplement: Supplementary file 1 [file healthcare-09-01151-s001.zip › healthcare-1339865-supplementary.pdf]

Appendix 1- Questionnaire

1.Socio-demographic data

Gender
Age
Civil state
Children (Y/N)
Age of younger son
Education
Workload (Full time/Part time /Unemployed/Student/Retired)

2. Emotions

Please indicate up to what extent you felt the following emotions during the last week, being 1 nothing and 5 a lot:

Calm	1	2	3	4	5
Nervous	1	2	3	4	5
Sad	1	2	3	4	5
Relaxed	1	2	3	4	5
Concerned	1	2	3	4	5

3. Teleworking

3.1. Is your professional activity compatible with teleworking?
3.2. Is teleworking conducted from home?
3.3. If you replied "Yes" to the previous question, in which part of the house are you typically working?
3.4. The equipment you are using for teleworking:
I already had it
I bought it
A friend/colleague borrowed me
Was provided by my employer
Other
3.5. The expenses with teleworking (internet, equipment, furniture) will be covered by the employer?
3.6. Are overtime hours reimbursed?
3.7. Do you need to communicate with colleagues in teleworking?
3.8. If you replied Yes, please indicate, in Column 1, all the tools that you use and, in column 2, the three more frequent ones:

	1	2
E-mail	☐	☐
Messenger	☐	☐
WhatsApp	☐	☐
Facebook	☐	☐
Microsoft Teams	☐	☐
Zoom Colibri	☐	☐
Skype	☐	☐
Slack	☐	☐
Linkedin	☐	☐
Others	☐	☐

If you replied "Others", please indicate which ones

3.9. What new tools did you start using during teleworking?

E-mail	☐
Messenger	☐
WhatsApp	☐
Facebook	☐
Microsoft Teams	☐
Zoom Colibri	☐
Skype	☐
Slack	☐
Linkedin	☐
Other	☐

3.10. How did you learn to use them?

Online Tutorial
Friends
Family
Training by employer/institution
Co-workers
Other

3.11. Did your professional daily routine changed?

3.12. If you replied "Yes", what has changed?

3.13. The workload in teleworking, when compared with regular work, is:

Higher
The same
Lesser

3.14. Have you enrolled in online courses to gain useful skills for your professional activity?

3.15. If you replied “ Yes”, what drove you?

Personal choice
Employer suggestion

3.16. How often do you communicate with your…

	+1x day	1x day	1 x week	x 1month
Close family	☐	☐	☐	☐
Enlarged family	☐	☐	☐	☐
Friends	☐	☐	☐	☐
Colleagues	☐	☐	☐	☐

3.17. Before the pandemic and the national emergency, how often did you communicate with your…

	+1x day	1x day	1 x week	1xmonth
Close family	☐	☐	☐	☐
Enlarged family	☐	☐	☐	☐
Friends	☐	☐	☐	☐
Colleagues	☐	☐	☐	☐

4. Technologies of communication and information

4.1. Have you been using communication technologies for leisure during the pandemic?
4.2. Did you use to shop online?
4.3. Currently, the frequency of online shopping increased?
4.3.1. If yes, of which goods?

5.Physical activity

5.1. Were you physically active before the pandemic?
5.1.1.If "yes", what type of physical activity? (Sports/Recreational)
5.1.2. If "Yes", how often?
Once a month
Once a week
One to three times a week
5.1.3. If " Yes", the physical activity took place:
Inside the house
Outside the house
5.1.4. The physical activity was done:
Alone
With one more person
In group
5.2. The physical activity changed since the beginning of the first lockdown?
Decreased
Did not change
Increased

5.3. Currently, how often do you engage in physical activity?

Once a month
Once a week
2 to 3 times a week
5 to 7 times a week
I do not

5.4. Currently, the physical activity takes place:

Inside the house
Outside the house

5.5. Currently, do you have a fixed routine for physical activity?

5.6. Currently, the physical activity is done:

Alone
With one person
In group

5.7.What is the type and quantity of physical activity required for your professional activity?

I am not employed (retired, unemployed)
I spend most of the time seated (office work)
I spend most of the time walking or standing but without significant effort
My work requires physical effort
My work requires intense physical activity

5.8.The type and quantity of physical activity has changed since the first lockdown?
